# Alternative flow cytometry strategies to analyze stem cells and cell death in planarians

**DOI:** 10.1002/reg2.53

**Published:** 2016-03-16

**Authors:** Tanuja Harshani Peiris, Marcos E. García‐Ojeda, Néstor J. Oviedo

**Affiliations:** ^1^ Department of Molecular and Cell Biology, School of Natural SciencesUniversity of CaliforniaMercedCalifornia95343USA; ^2^ Quantitative and Systems Biology Graduate ProgramUniversity of CaliforniaMercedCalifornia95343USA; ^3^ Health Sciences Research InstituteUniversity of CaliforniaMercedCalifornia95343USA

**Keywords:** Annexin‐V, cell death, DRAQ5, FACS, planarians

## Abstract

Planarians possess remarkable stem cell populations that continuously support cellular turnover and are instrumental in the regeneration of tissues upon injury. Cellular turnover and tissue regeneration in planarians rely on the proper integration of local and systemic signals that regulate cell proliferation and cell death. Thus, understanding the signals controlling cellular proliferation and cell death in planarians could provide valuable insights for maintenance of adult body homeostasis and the biology of regeneration. Flow cytometry techniques have been utilized widely to identify, isolate, and characterize planarian stem cell populations. We developed alternative flow cytometry strategies that reduce the number of reagents and the time of sample preparation to analyze stem cells and cell death in planarians. The sensitivity of these methods is validated with functional studies using RNA interference and treatment with  γ irradiation or stressful conditions that are known to trigger cell death. Altogether, we provide a community resource intended to minimize adverse effects during ex vivo studies of stem cells and cell death in planarians.

## Introduction

Planarian flatworms are attractive model organisms for studies of stem cell biology, mechanisms of disease, maintenance of body homeostasis, and tissue regeneration (Aboobaker 2011; Reddien 2013; Rink 2013). Planarians are able to regenerate missing parts in their body by activating adult somatic stem cells called neoblasts. Neoblasts are recognized as the only cells with proliferative capacity in planarians (Aboobaker 2011; Reddien 2013; Rink 2013). Thus, neoblasts constantly divide to support cellular turnover of adult tissues and to recreate missing or damaged parts after injury. Planarians possess a tightly regulated system capable of controlling neoblast proliferation in response to the presence of tissue damage and localized cell death that ensures body homeostasis. Significant research attention is focused on understanding intrinsic neoblast properties and the signals regulating neoblast behavior.

Over the last decade, an increasing number of methods and techniques have been developed to analyze neoblast regulation during cellular turnover and regeneration. One of these approaches involves flow cytometry experiments that allow classification of planarian cells based on different features. The original fluorescence activated cell sorting (FACS) protocol in planarians was developed by the laboratory of Kiyokazu Agata (Asami et al. 2002; Hayashi et al. 2006). This initial work demonstrated the feasibility of FACS to sort and characterize tissue‐specific cells through an *ex vivo* approach based on fluorescent markers that label DNA. The application of FACS analyses is commonly used in planarian research to evaluate DNA content, cell cycle dynamics, nuclear features, clonogenic potential, cell death, and the expression of markers related to neoblasts and differentiated tissues (Reddien et al. 2005; Oviedo & Levin 2007; Kang & Sánchez Alvarado 2009; Hayashi et al. 2010; Wagner et al. 2011; Moritz et al. 2012; Peiris et al. 2012; Shibata et al. 2012; Scimone et al. 2014; van Wolfswinkel et al. 2014; Zhu et al. 2015). FACS protocols are regularly coupled with modern molecular biology techniques and methods to characterize the complexity of neoblast subpopulations, loss‐of‐function phenotypes, pharmacological treatments, gene expression studies, and to develop genomic resources.

The classical work by Bardeen and Baetjer (1904) as well as Dubois (1949) demonstrated that planarian exposure to γ irradiation abolishes planarian regenerative properties and leads to lethality. This finding has proved quite useful to characterize neoblast function through FACS. Doses of γ irradiation, generally over 2000 rad, irreversibly eliminate neoblasts, which is followed by tissue loss (i.e., head regression), curling‐up of the ventral surface, and animal death in about 3 weeks (Wagner et al. 2011). Thus, γ irradiation is applied as a strategy to eliminate neoblasts and, through comparative analysis, elucidate their location in FACS profiles (Reddien et al. 2005; Hayashi et al. 2006). This approach identified three cell populations based on their sensitivity to γ irradiation: the irradiation sensitive X1 and X2 as well as the irradiation insensitive Xins (originally termed XIS). Cells within the X1 group contain proliferative neoblasts while cells in the X2 compartment are represented by a heterogeneous group including irradiation sensitive neoblasts, post‐mitotic progeny and other less characterized cell types. Differentiated cells mostly comprise the Xins component (Reddien et al. 2005; Hayashi et al. 2006; Eisenhoffer et al. 2008; Zhu et al. 2015).

Flow cytometry is also useful to analyze cell cycle and cell death parameters in planarians (Kang & Sánchez Alvarado 2009; Bender et al. 2012). The initial protocol for cell cycle analysis was introduced by the Sánchez Alvarado laboratory and has remained without changes for the most part (Kang & Sánchez Alvarado 2009). Results using annexin V−fluorescein isothiocyanate (FITC) and propidium iodide (PI) in planarians were briefly presented to demonstrate levels of cell death, but a detailed protocol of this procedure is not readily available (Bender et al. 2012). Altogether, flow cytometry protocols are essential components of the molecular repertoire to characterize neoblast function during cellular turnover and regeneration.

Hoechst stains are part of a family of nuclear staining dyes including Hoechst 33258, 33342, and 34580, which are common to almost all flow cytometry protocols in planarians (Asami et al. 2002; Reddien et al. 2005; Hayashi et al. 2006; Eisenhoffer et al. 2008; Scimone et al. 2010; Wagner et al. 2011; Hayashi & Agata 2012; Moritz et al. 2012; Romero et al. 2012; van Wolfswinkel et al. 2014). Hoechst dyes are membrane‐permeable and generally display lower toxicity than other nuclear markers such as DAPI (4′,6′‐diamidino‐2‐phenylindole). Hoechst 33342 is the most commonly used dye in the family, and can be excited around 355 nm by a UV light laser. When bound to DNA, it emits blue fluorescence around an emission maximum of 461 nm (BD Pharmigen 2015). This emission spectrum allows simultaneous FACS analysis with fluorescent markers with emission in the red and green spectra. Its spectral versatility and its low cost make Hoechst 33342 very attractive for flow cytometry studies. However, the use of Hoechst dyes also incorporates limitations that could interfere with experimental design (Durand & Olive 1982; Martin et al. 2005). For example, the Hoechst signal is quenched by simultaneous labeling with bromodeoxyuridine (BrdU), so for cell cycle analysis involving BrdU an alternative DNA marker such as DAPI is required (Crissman & Steinkamp 1987). Perhaps the most limiting consideration is the requirement of UV light or multiphoton laser to excite Hoechst dyes. Not all flow cytometer instruments incorporate UV lasers in their specifications. Moreover, the detrimental UV‐induced cellular damage, alterations in cell cycle, and cell death have been extensively documented in a variety of organisms including bacteria, plants, and animals (Stein et al. 1989; Hall et al. 1996; Cadet et al. 2005; Rastogi et al. 2010; Nawkar et al. 2013).

Here, we present an alternative flow cytometry protocol that reduces time of sample preparation and adopts the nuclear marker DRAQ5^TM^ (Smith et al. 1999, 2000) as a substitute for the traditional use of UV excitable staining in FACS and cell cycle studies. We also describe a detailed protocol to analyze cell death in planarians with annexin V and 7‐aminoactinomycin D (7‐AAD) staining that reliably monitors changes in cellular survival. The protocols described here are intended as a community resource to complement existing molecular tools and to minimize adverse effects by manipulation and staining with UV excitable markers.

## Results

### DRAQ5 as an alternative nuclear marker for FACS analysis in planarians

**Figure 1:**
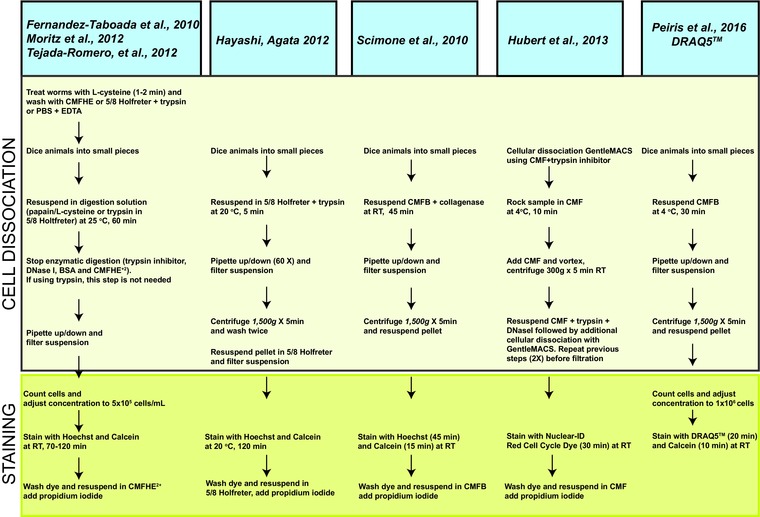
Schematic summary of recent FACS protocols in planarians. Representative variations of FACS protocols in planarians for the past 5 years are presented. The corresponding reference of the protocols is shown in the blue box and representative steps of the two main components of prepping samples before FACS analysis (i.e. whole planarian cellular dissociation and nuclear staining) are described within the two contiguous boxes. Notice the DRAQ5 and calcein‐AM protocol proposed does not involve enzymatic digestion or propidium iodide (PI) nuclear staining.

The initial FACS protocol adapted to planarian cells has been slightly modified over the years (Asami et al. 2002; Reddien et al. 2005; Hayashi et al. 2006; Oviedo & Levin 2007; Eisenhoffer et al. 2008; Fernandez‐Taboada et al. 2010; Pearson & Sánchez Alvarado 2010; Scimone et al. 2010; Hayashi & Agata 2012; Moritz et al. 2012; Peiris et al. 2012; Romero et al. 2012; Hubert et al. 2013). Nonetheless, all protocols involve two main preparatory steps before FACS analysis: (1) whole planarian cellular dissociation and (2) FACS staining procedures. These preparatory steps are preserved across different protocols and differences may apply mainly to the dissociation time and incubation with nuclear markers. Recently, Hubert et al., introduced modifications to the planarian dissociation and staining procedures (Hubert et al., 2013). Specifically, animal dissociation is carried out with additional equipment GentleMACS (Miltenyi Biotec) and cellular staining replaced Hoechst by the Nuclear–ID dye, which does not require UV‐excitation. The use of Hoechst and PI as DNA markers is standard to almost all protocols published to date (Asami et al. 2002; Hayashi et al. 2006; Hayashi & Agata 2012; Moritz et al. 2012; Romero et al. 2012). A schematic summary of the most recent versions of the protocol is included in Figure 1. The process of dissociation in most protocols involves physical disruption by dicing whole planarians with a razor blade followed by enzymatic digestion with trypsin, papain, or collagenase in a calcium−magnesium‐free (CMF) solution at either 4°C or room temperature. In some cases, planarian dissociation is preceded by treatment with mucolytic agents such as l‐cysteine hydrochloride monohydrate (Fernandez‐Taboada et al. 2010; Moritz et al. 2012; Romero et al. 2012). The Hoechst staining process varies among different protocols where some use staining for as little as 20 min (Reddien et al. 2005) or up to 120 min (Fernandez‐Taboada et al. 2010; Hayashi & Agata 2012). Altogether, the preparation time before flow cytometry in all these protocols is about 2 h and 30 min.

**Figure 2:**
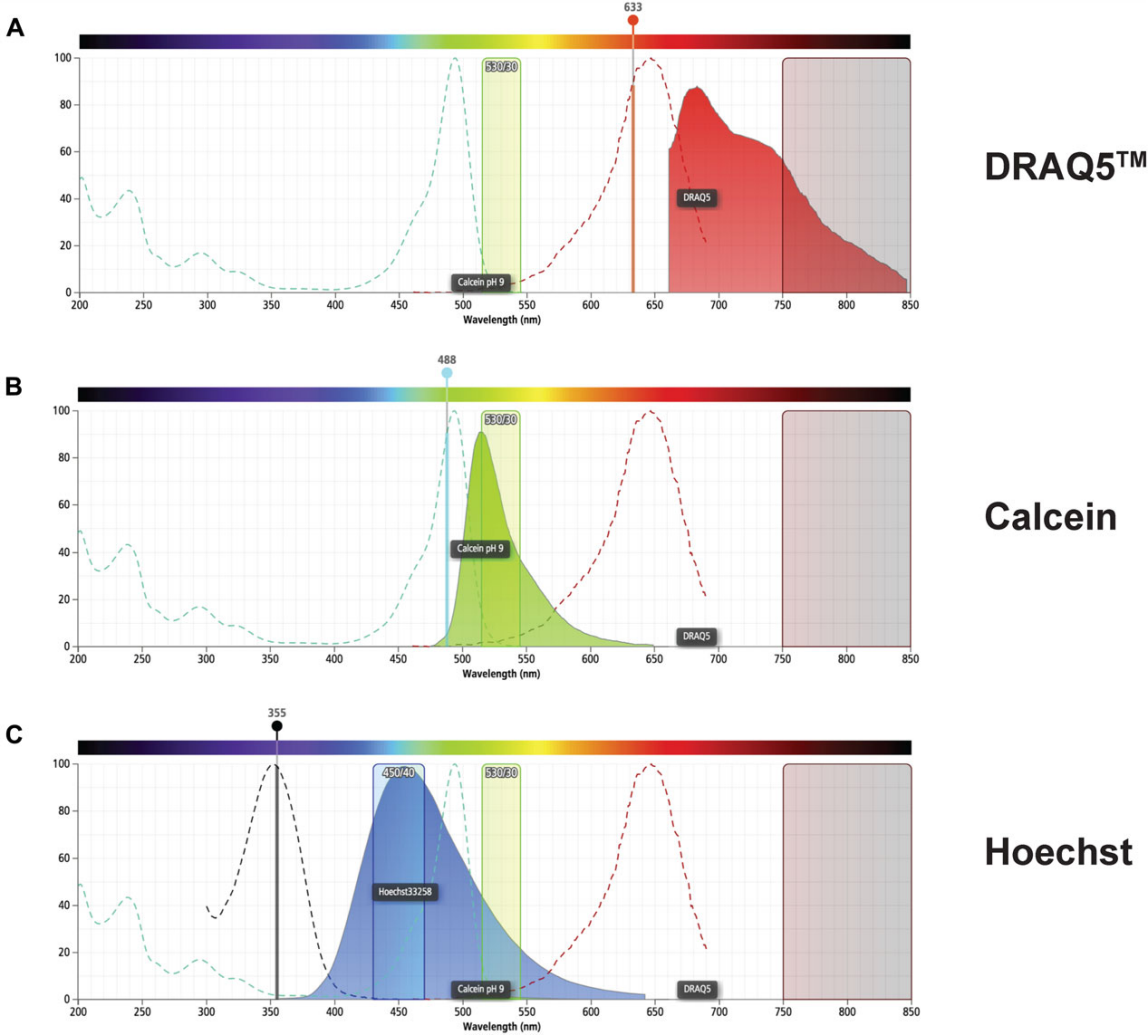
Comparison of the excitation and emission spectra of DRAQ5, calcein, and Hoechst. The absorption (dotted lines) and emission (solid, colored histogram) spectra for (A) DRAQ5, (B) calcein, and (C) Hoechst are shown. The collection channel filters for green (530/30) and far red (750LP) fluorescence are shown in all figures and (C) shows the collection channel for blue (450/40) fluorescence. The solid vertical lines show the 355 nm, 488 nm, and 633 nm lasers used to stimulate these fluorophores. Images were generated using BD Fluorescence Spectrum Viewer (http://m.bdbiosciences.com/us/s/spectrumviewer).

We have optimized an alternative FACS protocol that reduces two key aspects of the sample preparation: whole planarian dissociation and cellular staining (Fig. 1). First, our approach relies on whole planarian dissociation without mucolytic treatment or any other type of enzymatic digestion. We achieved high cellular yields and reproducible FACS results by mechanical disruption based on dicing worms with a razor blade followed by rocking in CMF solution for up to 30 min at 4°C (Fig. 1 and data not shown). Second, we use the nuclear dye DRAQ5 (1,5‐bis[2‐(di‐methylamino) ethyl] amino‐4,8‐dihydroxyanthracene‐9,10‐dione) instead of Hoechst for DNA staining (Smith et al. 1999, 2000). DRAQ5 (deep red fluorescing anthraquinone #5) is a far‐red fluorescent DNA marker, which is membrane permeable and can be used in live or fixed cells (Abcam® ab108410, Thermo‐Fisher 62251, Cell Signal 4084, eBioscience 65‐0880‐92). As in previous protocols, calcein staining is used to label living cells, replacing the need to use PI to distinguish live versus dead cells. The excitation and emission spectra for DRAQ5, Hoechst, and calcein are depicted in Figure 2.

#### Cellular dissociation for FACS analysis

In our experience, two planarians (∼6 mm length) yield about 1 million total cells. If dissociating 10 or fewer animals per sample, it is recommended to transfer the diced animals to a 15 mL conical tube. If more than 10 animals will be used in a sample, transfer the diced animals to a 50 mL conical tube.

Note that certain RNA interference (RNAi) phenotypes may render preparations more susceptible to cell death during manipulation. In those cases, it is advisable to use ∼20 animals per sample.

1. Prepare CMF medium plus 1% bovine serum albumin (BSA) (CMFB) without digestive enzymes and adjust the pH to 7.3 just before the experiment (Reddien et al. 2005). If keeping the CMF overnight, make sure to check and adjust its pH before use.
2. Place a 15 mL (or 50 mL) conical tube on ice and add 14 mL (or 45 mL) of CMFB medium. Keep tubes on ice at all times.
3. Planarian dissociation must be performed on a cold surface. Place 10−20 large planarians on a Petri dish (100 mm × 25 mm). Remove all water and add ∼2−3 mL of cold CMFB medium and gently wash the planarians. Note that adding the CMFB medium abruptly could create bubbles that potentially would reduce the yield of dissociation. After washing the animals, remove the CMFB medium completely since any leftover medium will reduce the dissociation efficiency. Use a Kimwipe to absorb the majority of the medium but leave some moisture behind around the worms.
4. Using a fresh razor blade, quickly dice the worms until their appearance is homogeneous with no recognizable tissue under the microscope. Note that cells should always be kept at low temperature with minimum time between steps.
5. Transfer the diced worms to a conical tube containing cold CMFB (from step 2). To increase the cellular yield, briefly rinse the Petri dish used to cut the animals by adding a few milliliters of cold CMFB to it. Transfer the rinse liquid to the same tube containing the diced tissue.
6. Gently rock the tube containing the dissociated planarians for 30 min at 4°C.

Note that during this incubation period the following preparatory steps could be performed:

- Setup of the flow cytometer instrument
- Preparation of the DRAQ5/CMFB solution
- Preparation of the calcein/CMFB solution (add dimethyl sulfoxide to the calcein stock and mix this calcein solution with CMFB; see below)

Note that dye mixes should always be kept cold and in the dark.

1. Strain the dissociated samples into a 50 mL conical tube using a 40 μm cell strainer.
2. Centrifuge the strained samples for 5 min at 1500 rpm at 4°C. Remove all the supernatant carefully. A cell pellet should be visible at the bottom of the tube.
3. Add 1−2 mL of ice‐cold CMFB medium and place the samples on ice.
4. Determine the cell number and viability using a hemocytometer and trypan blue. Note that ascertaining the cell number is essential to nuclear staining accuracy.

### Cellular staining for FACS analysis

The following protocol is optimized to stain a total of 1 million cells. If larger numbers of cells are going to be used for sorting purposes, scale up the total volume of dye mixtures accordingly. To sort X1, X2, and Xins cell populations we recommend increasing the number of cells to more than 20 million. If the sample is going to be used for cell population frequency or cell cycle analysis, we usually required about 2−3 million cells per experiment.

1. Add 1 μL of DRAQ5 to 1 mL of ice‐cold CMFB (5 μmol/L final concentration DRAQ5 per 1 million cells). Gently mix and place on ice in the dark.
2. Prepare a solution of calcein by adding 250 μL of dimethyl sulfoxide into the calcein stock tube (50 μg). Mix well.
3. Add 2 μL of the calcein mixture made in step 2 to 1 mL of ice‐cold CMFB (0.4 μg/mL final concentration calcein per 1 million cells). Gently mix and place on ice in the dark.
4. Place about 1 × 10^6^ cells into 1.5 mL centrifuge tubes. Spin the tubes for 5 min at 1500 rpm at 4°C. Discard the supernatant. A dark pellet should be visible at the bottom of the tube.
5. Gently resuspend the pellet with 500 μL of the DRAQ5 mixture (from step 1). The DRAQ5 final concentration that we recommend is 5 μmol/L per 1 million cells.
6. Incubate the tube at room temperature, in the dark, for 20 min.
7. Add 500 μL of the diluted calcein solution (from step 3) and mix gently.
8. Incubate the tube at room temperature, in the dark, for 10 min.
9. After incubation, place the tubes back on ice and keep them in the dark.
10. At this point the cells are ready for analysis or sorting. Pass the samples through the flow cytometer immediately.

Note that if an analysis or sorting takes more than 30 min, it is recommended to stagger the staining of different samples. Ideally each sample/condition would be exposed to the dyes for similar amounts of time.

### Cellular gating and FACS analysis

The stained, dissociated sample is heterogeneous, consisting of viable cells, cell aggregates, dead cells, and cellular fragments. To obtain reliable FACS data, it is necessary to analyze only viable, single cells, which in this case would be classified based on DNA content and their sensitivity to γ irradiation.

**Figure 3:**
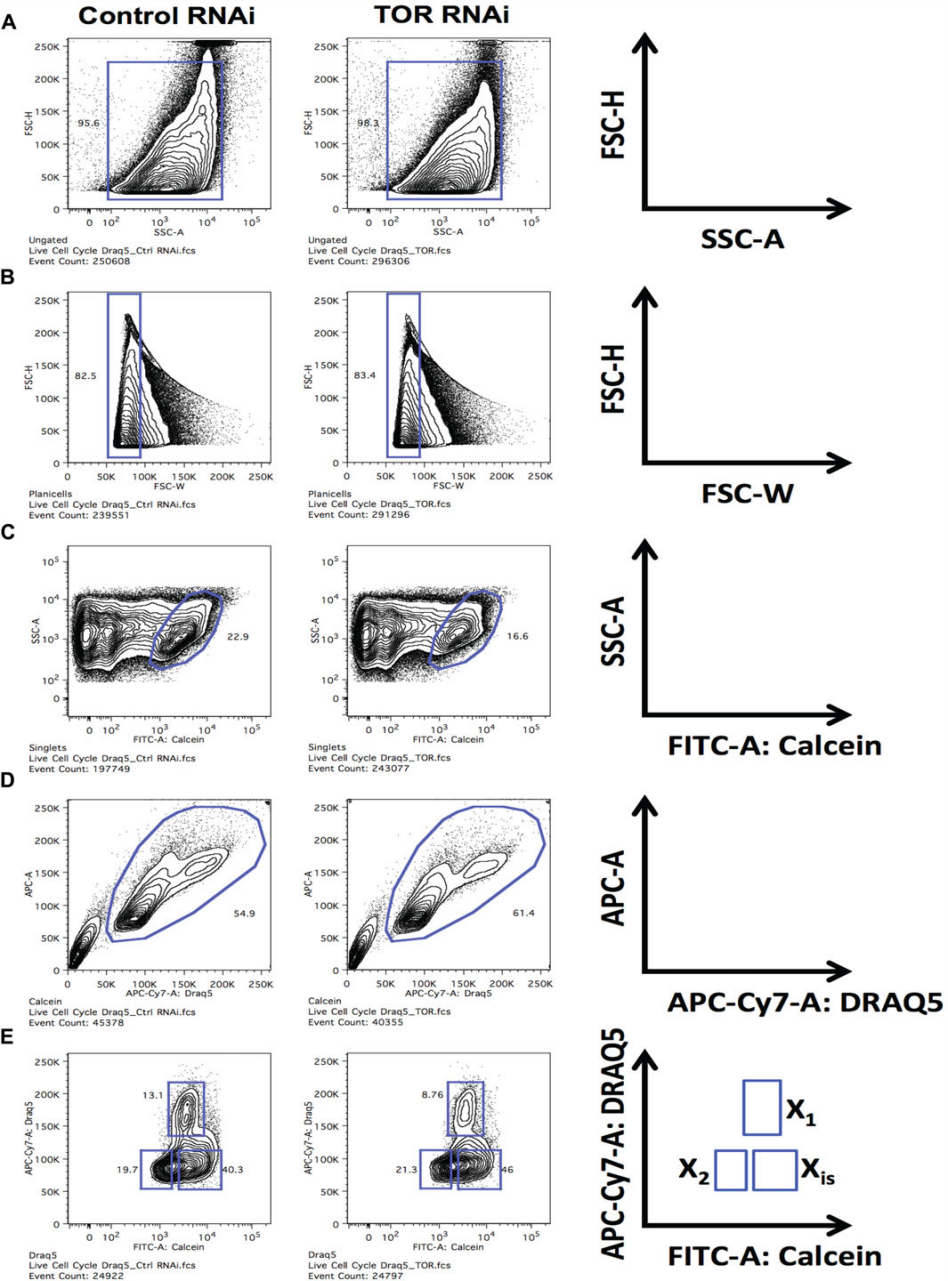
Sample gating strategy to identify planarian cell populations (X1, X2, and Xins). Planarian single cell suspension samples were obtained from five whole planarians and stained as described. To consider the representative number of events, a minimum of 250,000 events were collected per sample. This number would yield around 25,000 events in the last visualization. (A) FSC‐H (linear) and SSC‐A (log) parameters were analyzed and a gate (blue square) was placed over the majority of the cells. (B) Cells gated on (A) were visualized in FSC‐H versus FSC‐W. The narrow FSC‐W gate will contain single cells. (C) The single cells gated in (B) were visualized for SSC‐A versus FITC‐A:calcein (log). Calcein positive cells (oval blue gate) were selected to eliminate dead cells. (D) The calcein+ live cells (C) were visualized in APC‐A (linear) versus APC‐Cy7‐A:DRAQ5 (linear). This gate was done to eliminate calcein+ apoptotic cells that had less than 2n DNA content. The desired cells are inside the blue oval gate. (E) Cells gated in (D) were visualized in APC‐Cy7‐A:DRAQ5 (linear) versus FITC‐A:calcein (log). The X1, X2, and Xins populations are found in the blue square gates.

Below, we outline the sequential steps recommended to identify three subpopulations of planarian cells (X1, X2, and Xins) based on staining with DRAQ5 and calcein and their respective sensitivity to γ irradiation. The graphical illustration of this procedure (Fig. 3) is represented as a comparative profile between two pooled samples consisting of 10 worms each that were subjected to mock RNAi (control) and disruption with RNAi of the target of rapamycin (TOR) gene (Peiris et al. 2012).

1. Determine the size and granularity of the stained cellular mix by visualizing forward scatter height (FSC‐H, linear scale) and side scatter area (SSC‐A, log scale). This step will help to exclude cellular debris generated during the dissociation process (see gate outlined in blue in Fig. 3A). Cells maintain granularity and a complex architecture, which dust particles will not possess.
2. Select for single cells. The gated cell population in step 1 will contain single cells and cell aggregates (cell doublets, triplets, etc.). Selection of single cells, and accurate discrimination of doublets, is achieved by visualizing FSC‐H versus FSC width (FSC‐W) (Fig. 3B). Note that the cells generate a signal pulse as they pass through the instrument's laser beam. This pulse has a height and a width, giving information about the length of the cell passing through the beam. For accurate DNA measurements, this information is used to distinguish between single cells and doublets (Sharpless et al. 1975; Sharpless & Melamed 1976; Wersto et al. 2001).
3. Identify live cells based on calcein staining. Calcein‐AM is a membrane permeable viability dye that is retained by viable cells that use esterases to cleave its acetoxymethyl group. After cleavage, calcein will not be membrane permeable and will fluoresce. The 488 nm laser excites this dye and its peak emission is at 515 nm, such that it requires the green channel (530/30 filter) for its detection and collection. We use the SSC‐A and FITC‐A:calcein, both on a log scale, to identify cells that have cleaved calcein, which will denote the viable cell population in the sample (Fig. 3C). To effectively establish the gate, a single stain calcein control is needed.
4. Identify the pattern of the cells based on the DRAQ5 signal. The DNA bound DRAQ5 is excited by the 633 nm laser and emits far‐red fluorescence (> 650 nm). Therefore, we utilized the APC‐Cy7 channel, in linear scale, using the 750LP filter to collect its fluorescence (Fig. 3D). As opposed to Hoechst, DRAQ5 maintains spectral separation with calcein, which emits in the green spectrum and therefore does not require compensation (Fig. 2). The calcein+ live cells (C) can now be visualized in the APC‐A (linear) versus APC‐Cy7‐A:DRAQ5 (linear) channels. This oval blue gate eliminates calcein+ apoptotic cells that have less than 2n DNA content. Note that DRAQ5's broad emission range prevents it from being used together with PE‐Cy7, APC, APC‐Cy7 and Alexa Fluor 700 fluorophores.
5. Gate planarian cellular subpopulations by plotting DNA content versus live cell populations. This step denoting DRAQ5 on the ordinate axis and calcein signal on the abscissa allows visualization of X1, X2, and Xins populations (Fig. 3E). The cellular distribution is additionally confirmed by plotting side by side an irradiated sample displaying dramatic reduction of the irradiation sensitive X1 and X2 subpopulations (not shown).

**Figure 4:**
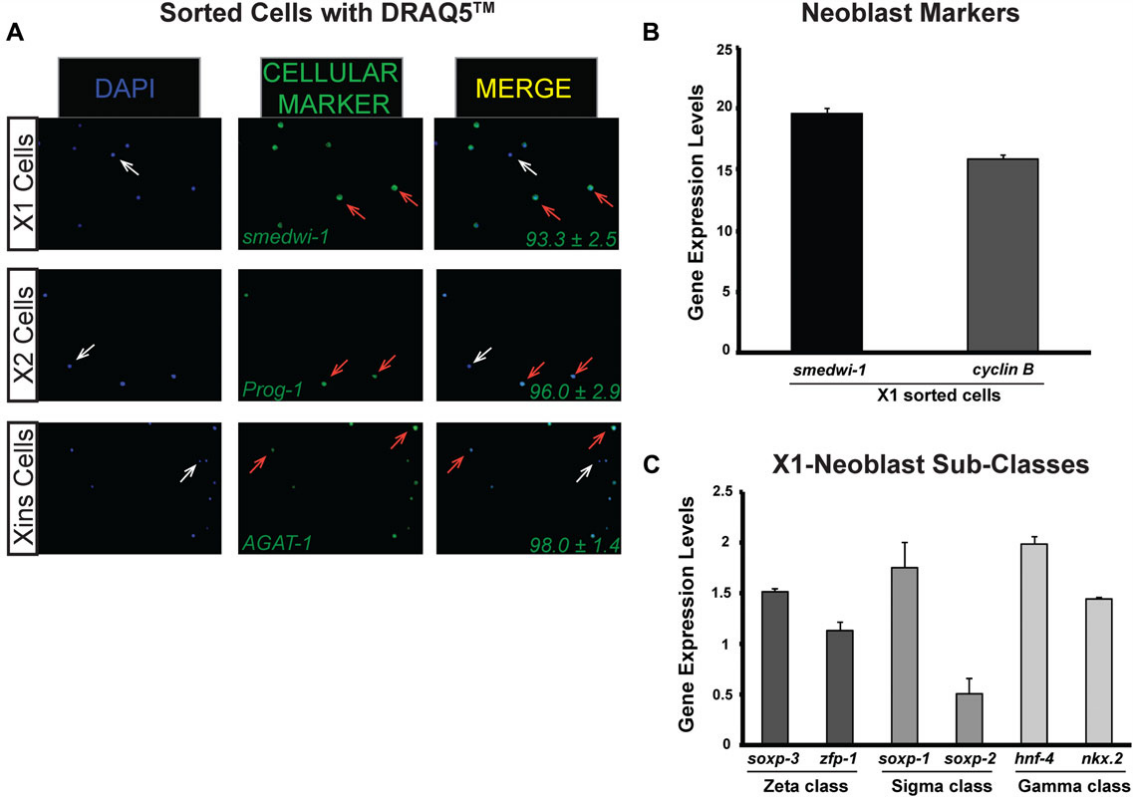
Cellular subpopulations isolated with DRAQ5 staining express neoblast and post‐mitotic markers. Planarian single cell suspension samples were obtained from five whole planarians and stained as described. (A) Fluorescent in situ hybridization using probes specific for X1, X2, and Xins cell populations. Cell nuclei were stained with DAPI (blue signal) and fluorescent anti‐sense probes (green signal) *smedwi‐1* (X1), *Prog‐1* (X2), and *AGAT‐1* (Xins). Arrows indicate either cells expressing the respective gene (orange) or cells that do not express the gene (white). The percentage of cells expressing each gene is represented at the bottom right corner of the respective merge image. Values represent the mean ± SD of three different samples obtained from the sorting of 10 dissociated planarians. (B), (C) Gene expression levels determined with qPCR using RNA extracted from X1 cells using neoblast specific probes and neoblast subclasses. qPCR analyses are from triplicate experiments; values represent the mean ± SD. Gene expressions are relative to the ubiquitously expressed clone *H.55.12e*.

In addition to the RNAi experiment, we also applied the gating and FACS analysis described above to analyze expression properties of sorted cells (X1, X2, and Xins). First, the expression of specific markers for each sorted cell population (e.g., X1, *smedwi‐1*; X2, *Prog‐1*; and Xins, *AGAT‐1*) was assayed through fluorescent in situ hybridization (Peiris et al. 2012). The percentage of cells expressing markers for the respective cell populations was consistent with previous results (Eisenhoffer et al. 2008), suggesting that our protocol is effective in sorting cells that express known markers for X1, X2, and Xins cells (Fig. 4A). The X1 cellular fraction includes proliferative neoblasts; thus we assayed through quantitative polymerase chain reaction (qPCR) the expression of the pan‐neoblast marker *smedwi‐1* and *Smed‐cyclinB* (*cyclinB* for short), which is specifically expressed within proliferative cells (Fig. 4B). Both *smedwi‐1* and *cyclinB* are highly expressed in the X1 group, confirming that proliferative cells are included. Recent studies revealed that the X1 group is heterogeneous and integrated by two prominent neoblast classes zeta and sigma (van Wolfswinkel et al. 2014). We found that the X1 cells sorted using DRAQ5 also express markers for each of the neoblast subclasses (Fig. 4C). Our findings indicate that the gating strategy and FACS analysis involving DRAQ5 staining are effective to study diverse planarian cell populations.

**Figure 5:**
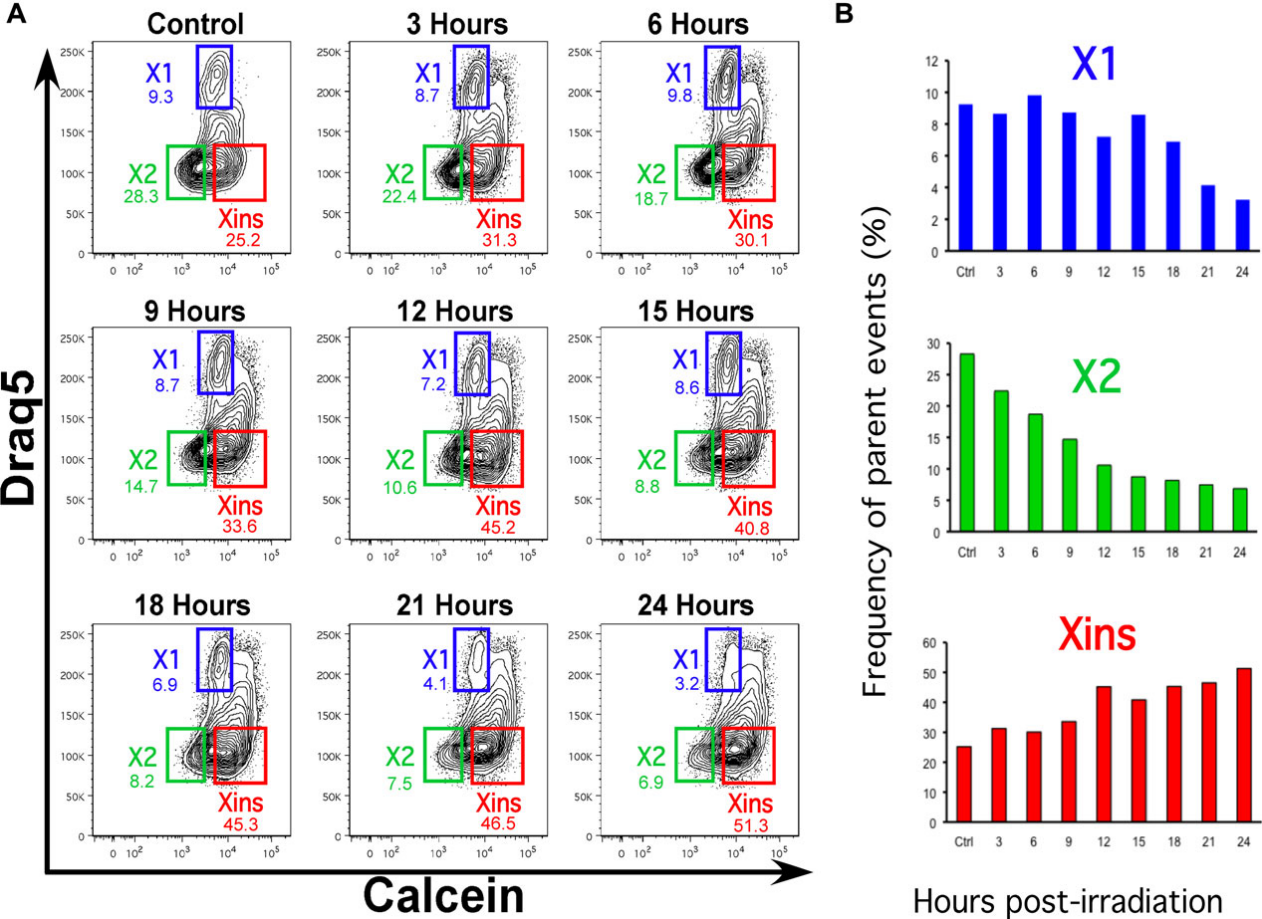
Effects of lethal irradiation on planarian cell populations. Planarians were exposed to a single lethal dose of γ irradiation (6000 rad) and a group of 10 animals were dissociated and cells stained with DRAQ5 and calcein every 3 h for 24 h. (A) FACS profiles with each cell population color‐coded blue, green, and red for X1, X2, and Xins, respectively. (B) The frequency of parent events for each individual cell population is presented in the bar graph to illustrate the changes over time.

We also assayed the sensitivity of DRAQ5 staining to determine changes in planarian cell populations after single lethal doses of γ irradiation (6000 rad). Briefly, planarians were irradiated and a group of 10 animals were dissociated every 3 h during 24 h. The changes for each planarian cell population (i.e., X1, X2, and Xins) are represented in Figure 5. Our results reveal that in the first 24 h there is a dramatic reduction of the radiation sensitive X1 and X2 cells, while the Xins population tends to increase after irradiation. These results demonstrate that the DRAQ5 staining and gating procedure described here are effective to record changes in cell populations after lethal irradiation.

## Flow cytometry protocol to analyze cell death in planarians

**Figure 6:**
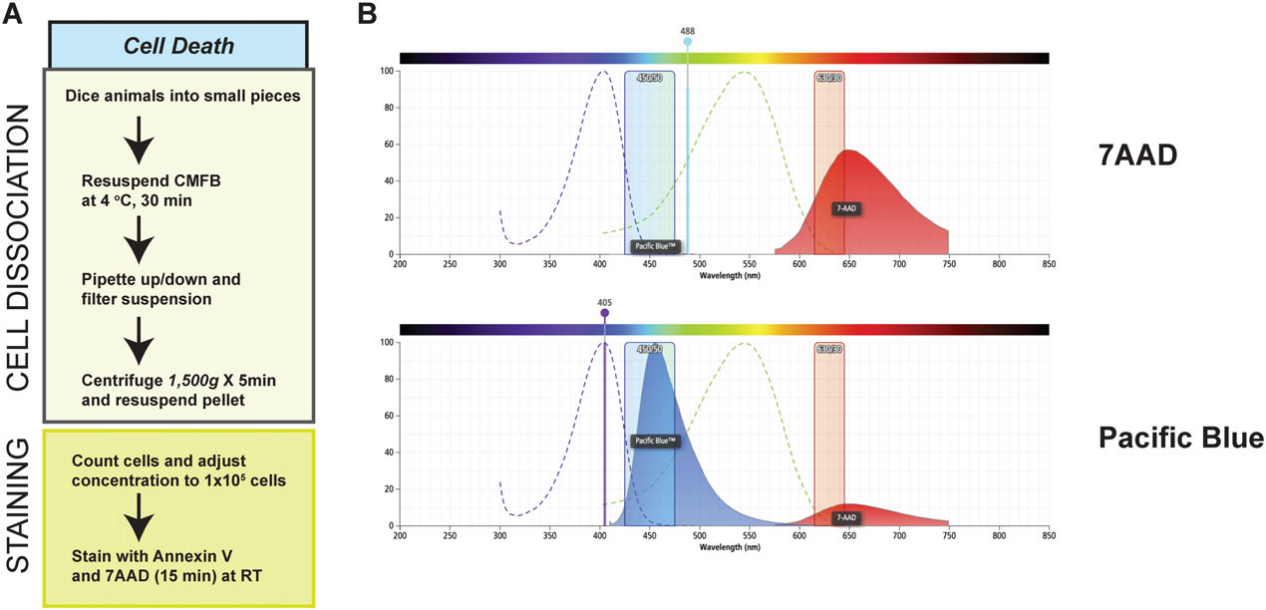
Prepping procedure for analysis of cell death and comparison of the excitation and emission spectra of 7‐AAD and Pacific Blue. (A) Representative summary of the cellular dissociation and staining procedures to analyze cell death. (B) The absorption (dotted lines) and emission (solid, colored histogram) spectra for 7‐AAD and Pacific Blue are shown. The collection channel filters for blue (450/40) and red (630/30) fluorescence are shown in all figures. The solid vertical lines show the 405 nm and 488 nm lasers used to stimulate these fluorophores. Images were generated using BD Fluorescence Spectrum Viewer (http://m.bdbiosciences.com/us/s/spectrumviewer).

Maintenance of homeostasis and tissue regeneration and patterning requires programmed cell death. Current immunostaining protocols using terminal deoxynucleotidyl transferase (TdT) dUTP nick‐end labeling (TUNEL) assay and caspase 3 shows systemic cell death in specific regions of the animal (Pellettieri & Sánchez Alvarado 2007; Pellettieri et al. 2010; Beane et al. 2013). However, these protocols pose some technical difficulties related to reproducibility in large size animals. The levels of cell death, however, could be easily surveyed by flow cytometry analysis using annexin V and 7‐AAD (Zimmermann & Meyer 2011; Zembruski et al. 2012), and we adopted these reagents to analyze cell death in planarians (Fig. 6A). Live cells contain phosphatidylserine (PS) phospholipids on the cytoplasmic leaflet of the plasma membrane. During apoptosis, PS is translocated and represented on the outer leaflet of the membrane, where it can be bound by fluorescently labeled annexin V protein. Thus, annexin V can be used to visualize cells that have initiated programmed cell death (Zimmermann & Meyer 2011). Although in our laboratory we utilized annexin V conjugated with the fluorochrome Pacific Blue, annexin V conjugated to other fluorochromes could be used as long as the fluorescence emission does not overlap with the emission of 7‐AAD.

Detection of apoptotic cells requires PI or 7‐AAD, which can be decided based on the optical specifications of the instrument available. PI has a broad range absorption spectrum and can be excited with the 488 nm laser. Its emission can be detected with the 630/22 filter. However, PI fluorescence overlaps significantly with the FL2 (575/26) and FL3 (670LP) channels (Boyd et al. 2008). To reduce these complications, we used 7‐AAD in our apoptotic assays. 7‐AAD is a DNA intercalating dye that is used to measure cell death (Zimmermann & Meyer 2011). This dye is excited by the 488 nm laser, its emission peak is at 650 nm and can be detected by the FL3 channel and, unlike PI, its fluorescence overlaps minimally with the FL2 channel (Fig. 6B, C). Furthermore, 7‐AAD does not leech from fixed, stained cells like PI, and its fluorescence remains stable for longer (Falzone et al. 2010).

**Figure 7:**
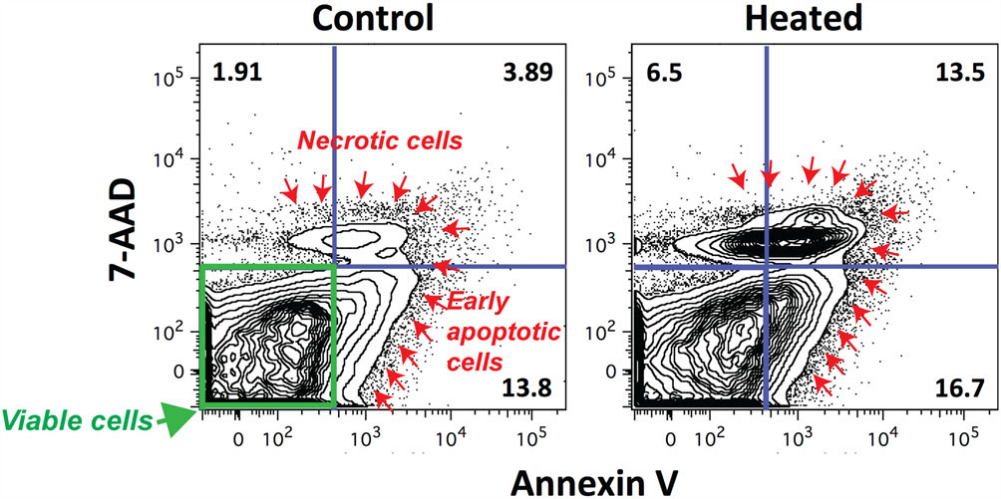
Distribution of living and dead cells stained with annexin V and 7‐AAD. Representative plot illustrating the distribution of viable cells (green label, lower left quadrant) and different stages of apoptotic cells (red arrows), early (lower, right quadrant) and late stage or necrotic cells (upper quadrants). Notice the increase in cell death after increasing the temperature to 60°C for 10 min. The analysis involves 1 × 10^5^ planarian cells from three worms.

For apoptosis analysis, planarian single cell suspensions were obtained as described above. To confirm that apoptotic planarian cells had the capability to stain efficiently with annexin V and 7‐AAD, we compared untreated cells to cells heated for 10 min at 60°C (Fig. 7). The initial FSC‐H versus SSC‐A and FSC‐H versus FSC‐W gates were performed as described in Fig. 3A and 3B, respectively, to concentrate the analysis in single cells. After this, the population was examined with annexin V on the *x* axis and 7‐AAD on the *y* axis, both parameters set in log phase (Fig. 7). The resulting plot displayed three populations: live cells (7‐AAD and annexin V negative, lower left quadrant), early apoptotic cells (7‐AAD negative and annexin V positive, lower right quadrant), and the late apoptotic or necrotic cells (7‐AAD and annexin V positive, upper right quadrant). Note the increase in percentage of dying cells in the heated sample (13.5%) compared to the unheated sample (3.9%).

## Discussion

Preserving cellular integrity is crucial for attaining reliable conclusions in ex vivo studies. Lengthy prepping time may influence cellular properties and affect the quality of the final results. Thus, our findings showing the feasibility of prepping dissociated planarian cells in half the time of current protocols provide a valuable alternative for studies of flow cytometry and subsequent analyses on isolated cells. Furthermore, whole planarian cellular dissociation in the absence of mucolytic agents or other types of enzymatic digestion probably contributes to preserve cellular integrity. Additionally, the protocol described here does not involve exposure to UV radiation, which is known to induce DNA damage (Stein et al. 1989; Rastogi et al. 2010; Nawkar et al. 2013). Altogether, we present protocols aimed at flow cytometry studies with fewer reagents, half of the prepping time, and without exposing cells to UV rays.

The protocols we describe for FACS analysis improve two main components of the prepping procedure, cellular dissociation and staining time, and are not dependent on the exclusive utilization of DRAQ5. For instance, whole worm dissociation could be performed without enzymatic digestion as we describe here and staining could be done using either Hoechst or DRAQ5 dyes. The proposed FACS protocol using DRAQ5 is reproducible and sensitive enough to determine changes in planarian cell populations after RNAi experiments and treatment with a lethal dose of γ irradiation. These results provide an effective alternative to FACS experiments using Hoechst. We advocate the use of DRAQ5, not only because it provides comparable results to Hoechst nuclear staining, but also because it has several advantages over Hoechst dyes. First, most bench top flow cytometry instruments come equipped with a 633 nm laser, while not all instruments have a UV light laser. Since DRAQ5 is excited by the 633 nm laser, this makes it a more accessible reagent for laboratories interested in performing flow cytometry studies of planarian stem cells and cell cycle. Moreover, DRAQ5 could be used in combination with BrdU staining to analyze cell cycle dynamics, a study that is not possible with Hoechst dyes (Crissman & Steinkamp 1987). The rapid membrane permeability and generalized intercalation of DRAQ5 to DNA reduce the staining time before FACS analysis and provide a more uniform DNA stoichiometric signal (Martin et al. 2005). Additionally, Hoechst dyes tend to preferentially bind to adenine and thymine (AT) rich regions within DNA, which could lead to characterization of partial regions of the genome and inconsistencies among cell populations (Durand & Olive 1982). Previous studies in cultured HeLa cells determined that DRAQ5 staining displays low bleaching behavior over time and it is less toxic than other DNA dyes, including TOPRO‐3, TOTO‐3, PI, and Hoechst 33258 (Martin et al. 2005). Our own empirical observations suggest that the 5 μM working concentration of DRAQ5 that we use in planarians has no apparent effects on cell cycle dynamics (unpublished observations). However, longer incubations with DRAQ5 (over 24 h) have been associated with disruption of G2/M phases of the cell cycle in vitro (Martin et al. 2005). Hoechst staining tends to diffuse through the cell over time, which may lead to potential pitfalls during the analysis of dividing and non‐dividing cells. Photobleaching with Hoechst is fast (within 1−2 min), while DRAQ5 stained cells maintain no bleaching behavior. Thus during flow cytometry sorts that span several hours, DRAQ5 will be a better staining candidate (Martin et al. 2005). Although the cost of DRAQ5 is higher than Hoechst, we believe the benefits from using a more stable DNA dye and a fast staining protocol compensate for the price differential of the reagent and are worth the investment. It is worth mentioning that the cost of a flow cytometry instrument with UV laser capability is probably much higher than the more affordable instrument without it. Additional experiments are needed to determine whether UV exposure during planarian cell FACS analysis triggers adverse cellular reactions, including DNA damage response, or affects cellular integrity. Thus, the use of DRAQ5 staining represents a viable alternative that would probably minimize the loss of vulnerable cell populations by reducing the time of *ex vivo* procedures.

We also present a simple protocol to evaluate apoptotic cell death in planarians. Tissue homeostasis and regeneration rely on opportune cell death and the current protocols available to analyze cell death in planarians (TUNEL and caspase‐3 immunostaining) have inherited experimental limitations due mostly to inconsistencies in whole‐mount staining. These studies are also done in cells that are fixed, while the FACS staining is done in live cells that can be utilized later for molecular or *in vivo* studies. The possibility of using our rapid whole planarian dissociation protocol followed by staining with annexin V and 7‐AAD represents an attractive alternative to analyze large groups of animals at once. Our protocol is highly sensitive, reproducible and could complement the spatial information obtained in whole‐mount staining for TUNEL and caspase‐3 immunostaining (Pellettieri & Sánchez Alvarado 2007; Pellettieri et al. 2010; Beane et al. 2013). Altogether, the protocols described here contribute to the arsenal of tools available to characterize the molecular basis of stem cell mediated tissue regeneration, adult tissue turnover and models of disease using planarians. These protocols are intended as community resources that would complement and facilitate in vivo analysis of stem cells and cell death in planarians.

## Materials and methods

### Planarian culture

The planarian species used in these experiments was *Schmidtea mediterranea* CIW4. *S. mediterranea* culture was maintained as previously described (Oviedo et al. 2008). Animals were starved for at least 1 week prior to experimentation.

### Reagents for DNA staining

Catalog numbers for the reagents used in DNA staining are as follows: Hoechst 33342, Life Technologies cat # H3570; DRAQ5, eBioscience cat # 65‐0880‐96; PI, Life Technologies cat # P3566; calcein‐AM, Life Technologies cat # C3100MP.

#### Calcium−magnesium‐free medium without digestive enzymes

The recipe for the CMF has been described previously (Reddien et al. 2005). Briefly, CMF plus BSA is 15 mmol/L HEPES, 400 mg/L NaH_2_PO_4_, 800 mg/L NaCl, 1200 mg/L KCl, 800 mg/L NaHCO_3_, 240 mg/L glucose, 1% BSA, pH 7.3.

### Flow cytometry data analysis

Samples were analyzed using either a BD LSRII flow cytometer (BD Biosciences) or sorted using a BD ARIAII flow cytometer (BD Biosciences), using BD FACSDiva™ software. Data analysis was performed with FlowJo software (TreeStar).

### Cell death analysis

Determination of cell death with flow cytometry was performed using 1 × 10^5^ dissociated planaria cells immersed in 100 μL of binding buffer (Bio Legend cat # 422201) and stained with 5 μL of annexin V−Pacific Blue (Bio Legend cat # 640918) and 5 μL of 7‐AAD viability staining solution (Bio Legend cat # 420404). Note that staining solution concentrations may vary so titrations are recommended. Labeled cells were incubated at room temperature for 15 min in the dark. The samples were immediately analyzed using an LSRII flow cytometer (BD Biosciences).

### Gamma irradiation of whole planarians

Lethally irradiated planaria provided reference cell populations to properly gate the radiation sensitive X1 and X2 cells, as previously described (Peiris et al. 2012). Briefly, planarians were exposed to a single lethal dose of γ irradiation (6000 rad) that is known to irreversibly eliminate neoblasts (Wagner et al. 2011). Seven days post‐irradiation, experimental and control animals were dissociated side by side as described above and used in flow cytometry studies.

## References

[reg253-bib-0001] Aboobaker, A.A. (2011). Planarian stem cells: a simple paradigm for regeneration. Trends Cell Biol 21, 304–311. 21353778 10.1016/j.tcb.2011.01.005

[reg253-bib-0002] Asami, M. , Nakatsuka, T. , Hayashi, T. , Kou, K. , Kagawa, H. and Agata, K. (2002). Cultivation and characterization of planarian neuronal cells isolated by fluorescence activated cell sorting (FACS). Zoological science 19, 1257–1265. 12499670 10.2108/zsj.19.1257

[reg253-bib-0003] Bardeen, C.R. and Baetjer, F.H. (1904). The inhibitive action of the Roentgen rays on regeneration in planarians. J. Exp. Zool. 1, 191–195.

[reg253-bib-0004] BD Pharmigen (2015). Hoechst 33342 solution. In: Technical Data Sheet. BD Pharmigen. San Jose, California, USA.

[reg253-bib-0005] Beane, W.S. , Morokuma, J. , Lemire, J.M. and Levin, M. (2013). Bioelectric signaling regulates head and organ size during planarian regeneration. Development 140, 313–322. 23250205 10.1242/dev.086900 PMC3597208

[reg253-bib-0006] Bender, C.E. , Fitzgerald, P. , Tait, S.W. , Llambi, F. , McStay, G.P. , Tupper, D. O. , Pellettieri, J. , Sánchez Alvarado, A. , Salvesen, G.S. and Green, D.R. (2012). Mitochondrial pathway of apoptosis is ancestral in metazoans. Proceedings of the National Academy of Sciences of the United States of America 109, 4904–4909. 22416118 10.1073/pnas.1120680109 PMC3324028

[reg253-bib-0007] Boyd, V. , Cholewa, O.M. and Papas, K.K. (2008). Limitations in the use of fluorescein diacetate/propidium iodide (FDA/PI) and cell permeable nucleic acid stains for viability measurements of isolated islets of Langerhans. Current trends in biotechnology and pharmacy 2, 66–84. 20814586 PMC2931281

[reg253-bib-0008] Cadet, J. , Sage, E. and Douki, T. (2005). Ultraviolet radiation‐mediated damage to cellular DNA. Mutation research 571, 3–17. 15748634 10.1016/j.mrfmmm.2004.09.012

[reg253-bib-0009] Crissman, H.A. and Steinkamp, J.A. (1987). A new method for rapid and sensitive detection of bromodeoxyuridine in DNA‐replicating cells. Experimental cell research 173, 256–261. 2960553 10.1016/0014-4827(87)90350-8

[reg253-bib-0010] Dubois, F. (1949). Contribution à l’étude de la migration des cellules de régénération chez les planaires dulcicoles. Bull. Biol. Fr. Belg. 83, 213–283.

[reg253-bib-0011] Durand, R.E. and Olive, P.L. (1982). Cytotoxicity, mutagenicity and DNA damage by Hoechst 33342. The journal of histochemistry and cytochemistry : official journal of the Histochemistry Society 30, 111–116. 7061816 10.1177/30.2.7061816

[reg253-bib-0012] Eisenhoffer, G.T. , Kang, H. and Sánchez Alvarado, A. (2008). Molecular analysis of stem cells and their descendants during cell turnover and regeneration in the planarian *Schmidtea mediterranea* . Cell stem cell 3, 327–339. 18786419 10.1016/j.stem.2008.07.002 PMC2614339

[reg253-bib-0013] Falzone, N. , Huyser, C. and Franken, D.R. (2010). Comparison between propidium iodide and 7‐amino‐actinomycin‐D for viability assessment during flow cytometric analyses of the human sperm acrosome. Andrologia 42, 20–26. 20078512 10.1111/j.1439-0272.2009.00949.x

[reg253-bib-0014] Fernandez‐Taboada, E. , Moritz, S. , Zeuschner, D. , Stehling, M. , Scholer, H. R. , Salo, E. and Gentile, L. (2010). Smed‐SmB, a member of the LSm protein superfamily, is essential for chromatoid body organization and planarian stem cell proliferation. Development 137, 1055–1065. 20215344 10.1242/dev.042564

[reg253-bib-0015] Hall, D.B. , Holmlin, R.E. and Barton, J.K. (1996). Oxidative DNA damage through long‐range electron transfer. Nature 382, 731–735. 8751447 10.1038/382731a0

[reg253-bib-0016] Hayashi, T. and Agata, K. (2012). A unique FACS method to isolate stem cells in planarian. Methods in molecular biology 879, 29–37. 22610551 10.1007/978-1-61779-815-3_2

[reg253-bib-0017] Hayashi, T. , Asami, M. , Higuchi, S. , Shibata, N. and Agata, K. (2006). Isolation of planarian X‐ray‐sensitive stem cells by fluorescence‐activated cell sorting. Development, growth & differentiation 48, 371–380. 10.1111/j.1440-169X.2006.00876.x 16872450

[reg253-bib-0018] Hayashi, T. , Shibata, N. , Okumura, R. , Kudome, T. , Nishimura, O. , Tarui, H. and Agata, K. (2010). Single‐cell gene profiling of planarian stem cells using fluorescent activated cell sorting and its "index sorting" function for stem cell research. Development, growth & differentiation 52, 131–144. 10.1111/j.1440-169X.2009.01157.x 20078655

[reg253-bib-0048] Hubert, A. , Henderson, J.M. , Ross, K.G. , Cowles, M.W. , Torres, J. and Zayas, R.M. (2013). Epigenetic regulation of planarian stem cells by the SET1/MLL family of histone methyltransferases. Epigenetics 8, 1–13. 10.4161/epi.23211 PMC3549883 23235145

[reg253-bib-0019] Kang, H. and Sánchez Alvarado, A. (2009). Flow cytometry methods for the study of cell‐cycle parameters of planarian stem cells. Dev Dyn 238, 1111–1117. 19322765 10.1002/dvdy.21928

[reg253-bib-0020] Martin, R.M. , Leonhardt, H. and Cardoso, M.C. (2005). DNA labeling in living cells. Cytometry. Part A : the journal of the International Society for Analytical Cytology 67, 45–52. 16082711 10.1002/cyto.a.20172

[reg253-bib-0021] Moritz, S. , Stockle, F. , Ortmeier, C. , Schmitz, H. , Rodriguez‐Esteban, G. , Key, G. and Gentile, L. (2012). Heterogeneity of planarian stem cells in the S/G2/M phase. The International journal of developmental biology 56, 117–125. 22450999 10.1387/ijdb.113440sm

[reg253-bib-0022] Nawkar, G.M. , Maibam, P. , Park, J.H. , Sahi, V.P. , Lee, S.Y. and Kang, C.H. (2013). UV‐induced cell death in plants. International journal of molecular sciences 14, 1608–1628. 23344059 10.3390/ijms14011608 PMC3565337

[reg253-bib-0023] Oviedo, N.J. and Levin, M. (2007). smedinx‐11 is a planarian stem cell gap junction gene required for regeneration and homeostasis. Development 134, 3121–3131. 17670787 10.1242/dev.006635

[reg253-bib-0024] Oviedo, N.J. , Nicolas, C.L. , Adams, D.S. and Levin, M. (2008). Establishing and maintaining a colony of planarians. CSH protocols 2008, pdb prot5053. 10.1101/pdb.prot5053 PMC10511150 21356691

[reg253-bib-0025] Pearson, B.J. and Sánchez Alvarado, A. (2010). A planarian p53 homolog regulates proliferation and self‐renewal in adult stem cell lineages. Development 137, 213–221. 20040488 10.1242/dev.044297 PMC2799157

[reg253-bib-0026] Peiris, T.H. , Weckerle, F. , Ozamoto, E. , Ramirez, D. , Davidian, D. , Garcia‐Ojeda, M.E. and Oviedo, N.J. (2012). TOR signaling regulates planarian stem cells and controls localized and organismal growth. J Cell Sci 125, 1657–1665. 22427692 10.1242/jcs.104711

[reg253-bib-0027] Pellettieri, J. and Sánchez Alvarado, A. (2007). Cell turnover and adult tissue homeostasis: from humans to planarians. Annu Rev Genet 41, 83–105. 18076325 10.1146/annurev.genet.41.110306.130244

[reg253-bib-0028] Pellettieri, J. , Fitzgerald, P. , Watanabe, S. , Mancuso, J. , Green, D. R. and Sánchez Alvarado, A. (2010). Cell death and tissue remodeling in planarian regeneration. Dev Biol 338, 76–85. 19766622 10.1016/j.ydbio.2009.09.015 PMC2835816

[reg253-bib-0029] Rastogi, R.P. , Richa , Kumar, A. , Tyagi, M.B. and Sinha, R.P. (2010). Molecular mechanisms of ultraviolet radiation‐induced DNA damage and repair. Journal of nucleic acids 2010, 592980. 21209706 10.4061/2010/592980 PMC3010660

[reg253-bib-0030] Reddien, P.W. (2013). Specialized progenitors and regeneration. Development 140, 951–957. 23404104 10.1242/dev.080499 PMC3583037

[reg253-bib-0031] Reddien, P.W. , Oviedo, N.J. , Jennings, J.R. , Jenkin, J.C. and Sánchez Alvarado, A. (2005). SMEDWI‐2 is a PIWI‐like protein that regulates planarian stem cells. Science 310, 1327–1330. 16311336 10.1126/science.1116110

[reg253-bib-0032] Rink, J.C. (2013). Stem cell systems and regeneration in planaria. Development genes and evolution 223, 67–84. 23138344 10.1007/s00427-012-0426-4 PMC3552358

[reg253-bib-0033] Romero, B.T. , Evans, D.J. and Aboobaker, A.A. (2012). FACS analysis of the planarian stem cell compartment as a tool to understand regenerative mechanisms. Methods in molecular biology 916, 167–179. 22914940 10.1007/978-1-61779-980-8_13

[reg253-bib-0034] Scimone, M.L. , Meisel, J. and Reddien, P.W. (2010). The Mi‐2‐like Smed‐CHD4 gene is required for stem cell differentiation in the planarian *Schmidtea mediterranea* . Development 137, 1231–1241. 20223763 10.1242/dev.042051 PMC2847463

[reg253-bib-0035] Scimone, M.L. , Kravarik, K.M. , Lapan, S.W. and Reddien, P.W. (2014). Neoblast specialization in regeneration of the planarian *Schmidtea mediterranea* . Stem cell reports 3, 339–352. 25254346 10.1016/j.stemcr.2014.06.001 PMC4176530

[reg253-bib-0036] Sharpless, T.K. and Melamed, M.R. (1976). Estimation of cell size from pulse shape in flow cytofluorometry. The journal of histochemistry and cytochemistry : official journal of the Histochemistry Society 24, 257–264. 1254921 10.1177/24.1.1254921

[reg253-bib-0037] Sharpless, T. , Traganos, F. , Darzynkiewicz, Z. and Melamed, M.R. (1975). Flow cytofluorimetry: discrimination between single cells and cell aggregates by direct size measurements. Acta cytologica 19, 577–581. 1108568

[reg253-bib-0038] Shibata, N. , Hayashi, T. , Fukumura, R. , Fujii, J. , Kudome‐Takamatsu, T. , Nishimura, O. , Sano, S. , Son, F. , Suzuki, N. , Araki, R. , et al. (2012). Comprehensive gene expression analyses in pluripotent stem cells of a planarian, *Dugesia japonica* . The International journal of developmental biology 56, 93–102. 22450997 10.1387/ijdb.113434ns

[reg253-bib-0039] Smith, P.J. , Wiltshire, M. , Davies, S. , Patterson, L.H. and Hoy, T. (1999). A novel cell permeant and far red‐fluorescing DNA probe, DRAQ5, for blood cell discrimination by flow cytometry. Journal of immunological methods 229, 131–139. 10556697 10.1016/s0022-1759(99)00116-7

[reg253-bib-0040] Smith, P.J. , Blunt, N. , Wiltshire, M. , Hoy, T. , Teesdale‐Spittle, P. , Craven, M.R. , Watson, J.V. , Amos, W.B. , Errington, R.J. and Patterson, L.H. (2000). Characteristics of a novel deep red/infrared fluorescent cell‐permeant DNA probe, DRAQ5, in intact human cells analyzed by flow cytometry, confocal and multiphoton microscopy. Cytometry 40, 280–291. 10918279 10.1002/1097-0320(20000801)40:4<280::aid-cyto4>3.0.co;2-7

[reg253-bib-0041] Stein, B. , Rahmsdorf, H.J. , Steffen, A. , Litfin, M. and Herrlich, P. (1989). UV‐induced DNA damage is an intermediate step in UV‐induced expression of human immunodeficiency virus type 1, collagenase, c‐fos, and metallothionein. Molecular and cellular biology 9, 5169–5181. 2557547 10.1128/mcb.9.11.5169-5181.1989 PMC363669

[reg253-bib-0042] Wagner, D.E. , Wang, I.E. and Reddien, P.W. (2011). Clonogenic neoblasts are pluripotent adult stem cells that underlie planarian regeneration. Science 332, 811–816. 21566185 10.1126/science.1203983 PMC3338249

[reg253-bib-0043] Wersto, R.P. , Chrest, F.J. , Leary, J.F. , Morris, C. , Stetler‐Stevenson, M.A. and Gabrielson, E. (2001). Doublet discrimination in DNA cell‐cycle analysis. Cytometry 46, 296–306. 11746105 10.1002/cyto.1171

[reg253-bib-0044] van Wolfswinkel, J.C. , Wagner, D.E. and Reddien, P.W. (2014). Single‐cell analysis reveals functionally distinct classes within the planarian stem cell compartment. Cell stem cell 15, 326–339. 25017721 10.1016/j.stem.2014.06.007 PMC4171737

[reg253-bib-0045] Zembruski, N.C. , Stache, V. , Haefeli, W.E. and Weiss, J. (2012). 7‐Aminoactinomycin D for apoptosis staining in flow cytometry. Analytical biochemistry 429, 79–81. 22796502 10.1016/j.ab.2012.07.005

[reg253-bib-0046] Zhu, S.J. , Hallows, S.E. , Currie, K.W. , Xu, C. and Pearson, B. J. (2015). A mex3 homolog is required for differentiation during planarian stem cell lineage development. eLife 4. 10.7554/eLife.07025 PMC4507787 26114597

[reg253-bib-0047] Zimmermann, M. and Meyer, N. (2011). Annexin V/7‐AAD staining in keratinocytes. Methods in molecular biology 740, 57–63. 21468968 10.1007/978-1-61779-108-6_8

